# Improved Clinical Outcomes With Early Anti-Tumour Necrosis Factor Alpha Therapy in Children With Newly Diagnosed Crohn’s Disease: Real-world Data from the International Prospective PIBD-SETQuality Inception Cohort Study

**DOI:** 10.1093/ecco-jcc/jjad197

**Published:** 2023-11-27

**Authors:** Renz C W Klomberg, Hella C van der Wal, Martine A Aardoom, Polychronis Kemos, Dimitris Rizopoulos, Frank M Ruemmele, Mohammed Charrout, Hankje C Escher, Nicholas M Croft, Lissy de Ridder, PIBD-SETQuality collaborative group, Ivan D Milovanovich, James J Ashton, Paul Henderson, Oren Ledder, Tim G J de Meij, Richard Hansen, Thalia Z Hummel, Katsuhiro Arai, Astor Rodrigues, Fiona Cameron, Sibylle Koletzko, Rafeeq Muhammed, Natalia Nedelkopoulou

**Affiliations:** Department of Pediatric Gastroenterology, Sophia Children’s Hospital, Rotterdam, The Netherlands; Department of Pediatric Gastroenterology, Sophia Children’s Hospital, Rotterdam, The Netherlands; Department of Pediatric Gastroenterology, Sophia Children’s Hospital, Rotterdam, The Netherlands; Pediatric Gastroenterology, Centre for Immunobiology, Blizard Institute, Barts and the London School of Medicine, Queen Mary University of London, London, UK; Department of Epidemiology, Erasmus Medical Centre, Rotterdam, The Netherlands; Department of Pediatric Gastroenterology, Université Paris Descartes, Sorbonne Paris Cité, Hôpital Necker Enfants Malades, Paris, France; Delft Bioinformatics Laboratory, Delft University of Technology, Delft, The Netherlands; Department of Pediatric Gastroenterology, Sophia Children’s Hospital, Rotterdam, The Netherlands; Pediatric Gastroenterology, Centre for Immunobiology, Blizard Institute, Barts and the London School of Medicine, Queen Mary University of London, London, UK; Department of Pediatric Gastroenterology, Sophia Children’s Hospital, Rotterdam, The Netherlands

**Keywords:** Biologics, inflammatory bowel disease, risk-stratification, early treatment

## Abstract

**Background and Aims:**

Treatment guidelines for paediatric Crohn’s disease [CD] suggest early use of anti-tumour necrosis factor alpha [anti-TNFα] in high-risk individuals. The aim is to evaluate the effect of early anti-TNF in a real-world cohort.

**Methods:**

Children with newly diagnosed CD were prospectively recruited at 28 participating sites of the international observational PIBD-SETQuality study. Outcomes were compared at 3 months, 1 and 2 years between patients receiving early anti-TNF [<90 days after diagnosis] and those not receiving early anti-TNF. Outcomes included sustained steroid-free remission [SSFR] without treatment intensification [specified as SSFR*] and sustained steroid-free mild/inactive disease without treatment intensification [specified as SSFMI*]. Penalised logistic regression model-based standardisation was applied to estimate the relative risks [RR] of early therapy on outcomes. RRs were estimated for high-risk and low-risk patients, based on presence of predictors of poor outcome [POPOs] and disease activity at diagnosis.

**Results:**

In total, 331 children (median age 13.9 years [IQR 12.2–15.3]) were enrolled, with 135 [41%] receiving early anti-TNF. At 1 year, patients on early anti-TNF had higher rates of SSFR* [30% vs 14%, *p* <0.001] and SSFMI* [69% vs 33%, *p* <0.001], with RRs of 2.95 [95% CI 1.63-5.36] and 4.67 [95% CI 2.46-8.87], respectively. At 1 year, the RRs for SSFMI* were higher, and statistically significant in high-risk patients, i.e. those with moderate/severe disease compared with mild/inactive disease at diagnosis (5.50 [95% CI 2.51-12.05] vs 2.91 [95% CI 0.92-9.11]), and those with any POPO compared with no POPO (5.05 [95% CI 2.45-10.43] vs 3.41 [95% CI 0.54-21.7]).

**Conclusion:**

In this cohort of children with newly-diagnosed CD, early anti-TNF demonstrated superior effectiveness in high-risk patients.

## Graphical Abstract

**Graphical Abstract:**
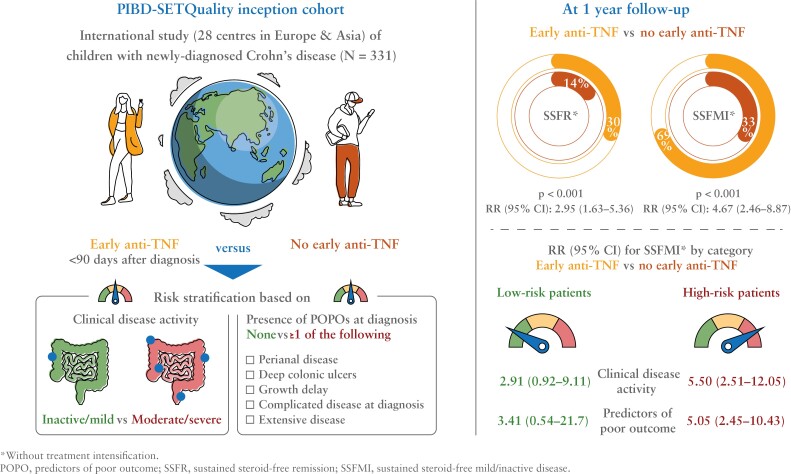

## 1. Introduction

The incidence of Crohn’s disease [CD] in children is rising dramatically.^1^ While most children with CD initially present with an inflammatory phenotype at diagnosis, 24–43% progress to a stricturing phenotype, and 14–44% to a penetrating phenotype.^2^ A treat-to-target strategy should be used, aiming to halt this disease progression, thereby preventing the need for surgery and reducing complications.

In 2021, the Selecting Therapeutic Targets in IBD [STRIDE-II] initiative proposed short- and long-term treatment targets for adult and paediatric patients with inflammatory bowel disease [IBD].^3^ The recommended short- and intermediate-term targets in children included achieving symptomatic clinical remission, normalisation of biomarkers such as C-reactive protein [CRP] and faecal calprotectin [FCP], and restored growth. Long-term targets involved endoscopic mucosal healing [MH] and normalised, health-related quality of life [HRQOL].

The optimal early therapeutic approach for achieving these treatment targets in paediatric CD is yet to be determined. Previous cohort studies in adults and children have shown the benefits of early anti-tumour necrosis factor alpha [anti-TNFα] use, leading to a shift in its utilisation towards earlier stages of the disease.^4–6^ However, these studies primarily focused on long-term outcomes unrelated to patient symptoms, such as disease progression to a stricturing and/or penetrating behaviour [B2 and/or B3 as per Paris classification].^7^ There is a need for clinical effectiveness studies that evaluate the short- and long-term effects of early treatment in CD patients in real-world scenarios.

As per the most recent ECCO-ESPGHAN guideline on the management of paediatric CD, early anti-TNF therapy is recommended solely for patients with specific predictors of poor outcomes [POPOs], such as severe growth delay, extensive disease, or deep colonic ulcers.^8^ The effects of early anti-TNF in these presumed ‘high-risk’ patients have not yet been studied. Furthermore, it remains unclear whether early anti-TNF may also be beneficial in ‘low-risk’ patients, e.g. patients without these predictors or those with mild disease at diagnosis. Gaining a better understanding of the effectiveness of early anti-TNF in different patient subgroups could contribute to the development of a clinical, risk-based treatment approach.

This study aimed to evaluate the effect of early anti-TNF, either as upfront therapy or initiated within 90 days after diagnosis, on treatment targets [clinical outcomes] proposed by the STRIDE-II initiative in children with CD, using real-world data from the prospective PIBD-SETQuality inception cohort.

## 2. Materials and Methods

### 2.1. Population description and eligibility

Data were obtained from the PIBD-SETQuality inception cohort, an ongoing, prospective, international, observational study. In this study, children aged 0–18 years with newly diagnosed IBD according to the revised Porto criteria were recruited at 28 paediatric IBD centres in Europe and Asia, and prospectively followed up according to the standard protocol reflecting usual clinical practice.^9,10^ Although the Paris classification for PIBD uses <17 years as a cut-off for defining age categories [A1a: 0-<10; A1b: 10–<17; A2: 17–40], the study protocol used 0–18 years, due to differing practices across multiple centres.^7^ Patients included in the inception cohort between January 1, 2017, and August 1, 2022, with a diagnosis of CD, were eligible for this study. All patients had to be therapy-naïve at diagnosis. The minimum duration of follow-up was 1 year. Patients who dropped out due to the transition to adult care, loss to follow-up, or study withdrawal before the 1-year mark, were excluded. Patients below 2 years of age were excluded because of the possibility of monogenic disease.

### 2.2. Data collection and variable definitions

#### 2.2.1. Clinical variables

For the current analyses, data were collected at baseline and 3, 6, 12, 18, and 24 months, and then annually up to the last follow-up, and included the following: age at diagnosis; sex; time to diagnosis [days between the onset of first symptoms and the date of initial diagnosis]; height; weight; body mass index [BMI]; clinical disease activity; endoscopic disease severity; therapy details [including start/stop date, dosing interval, and dosage]; Paris classification [disease location, behaviour, perianal disease]; specific local laboratory tests; local faecal calprotectin [FCP] level [see Supplementary Methods for details].^7^ As per study design, stool samples for FCP and blood samples for therapeutic drug monitoring were not mandatory and therefore not routinely assessed in each patient.

Clinical disease activity was scored by the weighted paediatric CD activity index [wPCDAI], further categorised into four groups [remission/inactive—mild—moderate—severe] according to validated cut-offs,^11^ or by the physician’s global assessment into one of these four categories in case of missing items of the wPCDAI. Endoscopic severity at diagnosis was scored by the simple endoscopic score for CD [SES-CD] and grouped into MH [SES-CD <3], mild endoscopic activity [SES-CD 3–9], and moderate-to-severe endoscopic activity [SES-CD ≥10].^12–14^ SES-CD could only be scored if the terminal ileum was intubated during endoscopy.

Patients were managed according to the discretion of the treating physicians, not by standardised protocols. Decisions for treatment intensification were made based on clinical and laboratory data in addition to therapeutic drug monitoring results [anti-TNF trough levels and anti-drug antibodies] if available.

#### 2.2.2. Predictors of poor outcome [POPOs]

The ESPGHAN paediatric CD guideline has identified several POPOs: perianal disease, deep colonic ulcers, extensive disease, growth delay, and complicated disease.^8^ Patients exhibiting one or more POPOs were considered high-risk patient and those without any POPO were considered low-risk patients. In this study, the SES-CD ‘size of ulcer’ sub-score was used to classify ‘large’ or ‘very large’ ulcers as deep colonic ulcers. Extensive disease was defined as macroscopic ileocolonic disease [L3] along with proximal disease [L4a and/or L4b], regardless of the severity observed during endoscopy. Growth delay was evaluated by standardising growth parameters to Z-scores based on calendar age and the WHO Child Growth Standards.^15^ Growth delay was defined as a height for age [HFA] Z-score < -1.5. In accordance with the ESPGHAN guideline, the POPO ‘complicated disease’comprised both patients with B1 inflammatory disease with narrowing, but without prestenotic dilatation, as well as with a B2 and/or B3 phenotype forming the POPO ‘complicated disease’ category.

### 2.3. Outcome evaluation

#### 2.3.1. Early anti-TNF therapy

To analyse our data, study participants were stratified into two groups: the early anti-TNF therapy group and the no early anti-TNF therapy group. Early anti-TNF was defined as infliximab or adalimumab administered within 90 days after the initial diagnosis, either as upfront biologic therapy or as early intensified treatment.

#### 2.3.2. Outcome definitions

The primary outcome assessed at 1 year was sustained steroid-free remission [SSFR] without treatment intensification [specified as SSFR*]. This was defined as being in continuous clinical remission without steroids and without treatment intensification from 3 months after diagnosis up to the time point of assessment. Secondary outcomes evaluated at 1 year included clinical remission, normal CRP remission [NCR: remission with CRP <0.5 mg/dl], normal FCP remission [NFR: remission with FCP <250 μg/g], steroid-free mild or inactive disease [SFMI: mild or inactive clinical disease activity without use of steroids], sustained SFMI [SSFMI: being in continuous SFMI between 3 months and the time point of assessment], SSFMI without treatment intensification [specified as SSFMI*], and MH. Treatment intensification was defined as escalation to second-line biologic therapy [eg, ustekinumab, vedolizumab] in the early anti-TNF group, escalation to any biologic agent in the no early anti-TNF group, or need for IBD-related luminal surgery, prior to the time point of outcome assessment in any group. In-class switch [ie, infliximab to adalimumab or vice versa] and dose intensification were not considered treatment escalation. We also compared the mucosal inflammation noninvasive index [MINI-index], a non-invasive tool to predict the degree of endoscopic mucosal inflammation in children with CD, which can be used as a proxy for mucosal healing [MH] [see Supplementary data].^14^

Short-term outcomes to early therapy were evaluated at 3 months. Patients with a ≥50% decrease in FCP compared with baseline FCP or with an FCP <250 μg/g were considered FCP-responders. Small and moderate response were defined as a decrease in wPCDAI of >17.5 points or ≥1 PGA category compared with baseline [small response], or >37.5 points or ≥2 PGA categories compared with baseline [moderate response].

#### 2.3.3. Growth and quality of life

Other STRIDE-II treatment targets include normalised growth and normalised HRQOL. To evaluate growth, we compared changes in HFA and BMI Z-scores [presented as ΔZ-scores] between baseline, and 1 or 2 years, which were calculated using the anthropometric reference data from the WHO Child Growth Standards, using the *zscorer* package in R.^15,16^

HRQOL was assessed using the IMPACT-III questionnaire, a validated 35-item IBD-specific measure of HRQOL for children [range 0–100], and the EQ-5D-5L questionnaire, a generic HRQOL instrument which includes the visual analogue scale [VAS], recording the patient’s self-rated health [range 0–100] [see Supplementary data].^17,18^ Changes in IMPACT-III and EQ-5D VAS scores were presented as Δ-scores.

### 2.4. Missing data and multiple imputation

Missing data for variables that were included as covariate in regression models were imputed. We assumed the POPOs perianal disease and complicated disease to be negative in case of missing data. The other data were determined to be missing at random. To address missing data, we performed multiple imputations by the chained equations, using the R package *mice (*R statistical software version 4.2.2 [R Development Core Team, 2012]). The imputation model included the covariates used in our regression model [imputed variables] and variables that were possibly related to these regression model covariates [auxiliary predictor variables], including age at diagnosis, sex, wPCDAI, disease activity [categorised], upper and lower SES-CD, disease location, upper gastrointestinal tract disease location, all POPOs, and all laboratory data, including FCP. A total of 30 imputed datasets were generated, using 10 iterations. The results from the imputed datasets were pooled using Rubin’s rules to obtain the final estimates and their corresponding standard errors.

### 2.5. Statistical analysis

#### 2.5.1. Descriptive statistics

Continuous data were presented as mean ± standard deviation, or median [interquartile range] depending on the probability distribution, and compared using the unpaired two-sample t test or Mann–Whitney U test, as appropriate. Paired continuous data were analysed using the paired sample t test. Frequencies of categorical variables were compared using either the chi square test or Fisher’s exact test, as appropriate.

#### 2.5.2. Penalised logistic regression

To evaluate the effect of early anti-TNF on outcomes, regression models were fitted while accounting for potential confounding effects. We included in our model baseline covariates based on clinical relevance and prior literature, selecting covariates that were found to be possibly associated with chronically active disease or complicated disease course, such as the need for surgery or B2/B3 disease.^19^ These covariates were age at diagnosis, sex, moderate-to-severe disease activity, CRP level, and all five POPOs. Variables with too many missing data [>50%], such as FCP, or data assumed to be not missing at random, such as SES-CD, were not included as these could not be imputed. Missing values for other confounders were imputed following the previously described method.

First, a penalised binary logistic regression model was fitted to estimate the conditional [on the confounders] odds ratio [OR] of early anti-TNF on the primary outcome, while adjusting for baseline covariates. We fitted a penalised logistic regression model using the ridge penalty—Bayesian prior distribution. This method simultaneously estimates the coefficients for predictor variables while imposing a penalty on the quadratic values of the coefficients, thereby encouraging shrinkage towards zero. This approach enabled the inclusion of a larger number of covariates in the model while shrinking the coefficients of less important predictors.

#### 2.5.3. Regression standardisation

Next, we aimed to examine the causal effect of early anti-TNF on the outcomes, by applying the method of regression standardisation with bootstrapping. Standardisation allows estimation of marginal measures of association, such as crude relative risks [RRs] or crude ORs, in observational studies, by emulating a conditional randomised experiment.^20,21^ If the measured confounders are sufficient for confounding control, then the marginal association measures can be interpreted as population causal effects.

First, we fitted the penalised logistic regression model of our outcome, using the same covariates as previously described. Subsequently two datasets were created, in which the therapy was either early anti-TNF or no early anti-TNF for all subjects, while the distribution of the covariates was fixed. Next, we obtained the individual predictions of the outcome for each patient in both datasets. Finally, we calculated the crude OR and RR. To obtain the estimate and its variance per imputed dataset, we applied bootstrapping. For each bootstrap sample, the model was fitted, and the resulting coefficients were estimated using the maximum likelihood method with the ridge penalty incorporated. This process was repeated 1000 times to generate a distribution of model estimates. The analyses were then run for each imputed dataset and the results were combined using Rubin’s rules.

#### 2.5.4. Evaluating a risk-stratified approach using subgroup analyses

In an aim to compare the effectiveness of early anti-TNF between presumed ‘low-risk’ and ‘high-risk’ patients, we conducted an analysis focusing on specific patient subgroups: those with mild or inactive clinical disease activity [low-risk] vs moderate-or-severe clinical disease activity at diagnosis [high-risk], and those with no POPO [low-risk] vs those with one or more POPOs [high-risk]. To ensure an adequate effective sample size for our regression model, allowing us to incorporate the same covariates consistently throughout our analysis, we used SSFMI* as the outcome of interest for these subgroup analyses, considering that only a subset of the dataset was employed. Stratifying patients by risk would reduce the effective sample size too much to yield valid causal estimates of the primary outcome, SSFR*.

#### 2.5.5. Evaluating the time-independent effect of early therapy

In an effort to eliminate the time-dependent effect of early therapy on sustaining remission, we assessed rates of SSFR* and SSFMI* at 1 and 2 years for those patients that were in SFR and SFMI at 3 months, respectively, and compared these by early therapy group.

All computations were conducted using IBM SPSS Statistics version 27.0 [IBM, Armonk, NY] or R Statistical Software. A two-sided *p*-value <0.05 was considered statistically significant.

### 2.6. Ethics statement and informed consent

The PIBD-SETQuality study protocol was first approved by the institutional review board of the Erasmus Medical Centre Rotterdam [MEC-2016-321], and subsequently by the institutional review boards of all participating centres. Written informed consent was provided by caregivers or legal guardians of all study participants and by patients themselves, as appropriate according to national regulations.

## 3. Results

### 3.1. Study population

In total, 417 children with CD met the eligibility criteria, of whom 332 [80%] were followed up through 1 year. One patient was excluded because of missing data for early anti-TNF use. Patients were recruited at 28 centres in Europe [*n* = 23], Asia [*n* = 2], and Israel [*n* = 5] [Supplementary Table 1]. Most patients were recruited in the UK [*n* = 170; 51%] and The Netherlands [*n *= 99; 30%]. Nine [3%] patients were diagnosed with very-early-onset IBD [range 3.2–5.9 years].

Of 331 patients [41%], 135 received early anti-TNF (91 [67%] infliximab, 44 [33%] adalimumab), of whom 64 [47%] received upfront anti-TNF while being therapy-naïve [Table 1]. Median time from diagnosis to initiation of early anti-TNF was 28 days [IQR 7–50], which was significantly shorter for infliximab (18 days [IQR 6–43]) as compared with adalimumab (44 days [IQR 16–61]], *p* = 0.02). Patients with no early anti-TNF were mostly treated with exclusive enteral nutrition (*n *= 136 [69%]] or steroids [*n* = 41 [21%]) as induction therapy.

**Table 1:** Patient and disease characteristics at diagnosis of the paediatric Crohn’s disease study population, stratified by early anti-TNF use

Characteristics	Early anti-TNF therapy [*n *= 135; 59%]	No early anti-TNF therapy [*n *= 196; 41%]	Total [*n* = 331]	*p*-value
Age in years, median [IQR]	13.9 [12.2–15.3]	13.2 [10.8–15.1]	13.7 [11.4–15.2]	**0.03**
Male sex, *n* [%]	88 [65]	116 [59]	204 [62]	0.32
First-degree relative with IBD, *n* [%]	23 [17]	42 [21]	65 [20]	0.40
Ethnicity, *n* [%]				0.73
White	87 [73]	120 [67]	207 [70]	
Asian	15 [13]	23 [13]	38 [13]	
Black	3 [3]	5 [3]	8 [3]	
Hispanic/Latino	1 [1]	1 [1]	2 [1]	
Mixed/other	13 [11]	29 [16]	42 [14]	
Time to diagnosis in days, median [IQR]	141 [76–365]	159 [83–250]	153 [79–285]	0.85
BMI Z-score, mean [SD]	-1.21 [1.48]	-0.79 [1.50]	-0.96 [1.51]	**0.013**
Disease location,^a^*n* [%]				**<0.001**
L1 isolated ileal	21 [15]	61 [32]	82 [25]	
L2 isolated colonic	16 [12]	40 [21]	56 [17]	
L3 ileocolonic	95 [71]	85 [45]	180 [56]	
L4 isolated upper disease	2 [2]	3 [2]	5 [2]	
Upper gastrointestinal tract involvement^a^, *n* [%]				0.33
L4a	61 [45]	84 [44]	145 [45]	
L4b	12 [9]	10 [5]	22 [7]	
L4ab	0 [0]	3 [2]	3 [1]	
No upper disease	62 [46]	92 [49]	154 [48]	
Perianal disease,^a^*n* [%]	46 [34]	19 [10]	65 [20]	**<0.001**
Disease behaviour,^a^*n* [%]				0.21
B1	112 [83]	174 [89]	286 [87]	
B2	12 [9]	15 [8]	27 [8]	
B3	9 [7]	5 [3]	14 [4]	
B2B3	2 [2]	1 [1]	3 [1]	
Disease activity, *n* [%]				**0.006**
None/inactive	4 [3]	11 [6]	15 [5]	
Mild	27 [20]	67 [35]	94 [29]	
Moderate	51 [38]	66 [34]	117 [36]	
Severe	52 [39]	48 [25]	100 [31]	
Moderate-to-severe disease activity, *n* [%]	103 [77]	114 [59]	217 [67]	**0.002**
wPCDAI, mean [SD]	51 [22]	44 [21]	47 [21]	**0.005**
Endoscopic severity,^b^*n* [%]				0.13
Mucosal healing	3 [3]	8 [6]	11 [5]	
Mild endoscopic inflammation	15 [17]	38 [27]	53 [23]	
Moderate-to-severe endoscopic inflammation	73 [80]	897 [68]	170 [73]	
SES-CD score, median [IQR]	12 [7–18]	15 [11–22]	13 [8–19]	**0.009**
Induction therapy, *n* [%]				**<0.001**
Exclusive Enteral Nutrition	47 [35]	136 [69]	183 [55]	
Corticosteroids	19 [14]	41 [21]	60 [18]	
5-aminosalicylates	1 [1]	9 [5]	10 [3]	
Upfront anti-TNF^c^	64 [47]	n/a	64 [19]	
None/other^d^	4 [3]	10 [5]	14 [4]	
Steroid use within first 3 months, *n* [%]	35 [26]	74 [38]	109 [33]	**0.024**
IMM use within first 3 months, *n* [%]	110 [82]	128 [65]	238 [72]	**<0.001**

Missing values for each variable: age in years 0; male sex 0; first-degree relative with IBD 0; ethnicity 34; days to diagnosis 12; BMI Z-score 12; disease location 8; upper gastrointestinal tract involvement 7; perianal disease 10; disease behaviour 1; disease activity 5; wPCDAI 77; endoscopic severity 97; SES-CD score 97; induction therapy 0; steroid use within first 3 months 1; IMM use within first 3 months 1.

SES-CD, Simple Endoscopic Score for Crohn’s disease; anti-TNF: anti-tumour necrosis factor alpha; IMM: immunomodulator [methotrexate, azathioprine, 6-mercaptopurine, thioguanine]; IQR: interquartile range; SD: standard deviation; wPCDAI: weighted paediatric Crohn's disease activity index.

^a^As per Paris classification.

^b^Based on SES-CD reference values according to MINI-index paper.

^c^Most [*n* = 41] of these patients had one or more clear indications to start upfront anti-TNF, including active perianal disease [*n *= 19], B2/B3 disease at diagnosis [*n *= 18], inclusion in the TISKids trial [*n* = 7], active EIMs [*n* = 8]. Patients with upfront anti-TNF could also have one or multiple predictors of poor outcome, such as growth delay [*n* = 13], extensive disease [*n* = 30], deep colonic ulcer [*n *= 22]. Three patients did not have any predictor of poor outcome or other clear indication for upfront anti-TNF.

^d^Five dietary therapy [Crohn’s disease exclusion diet with partial enteral nutrition], three antibiotics, three monotherapy with an immunomodulator, two no therapy, one unknown.

At baseline, patients with early anti-TNF had moderate-to-severe disease activity significantly more often [77% vs 59%, *p* = 0.002], higher endoscopic severity scores [median SES-CD 15 [IQR 11–22] vs 12 [IQR 7–18], *p* = 0.009], and higher serum inflammatory markers (median CRP 25 [IQR 13–51] vs 14 [IQR 4–40], *p* <0.001; median erythrocyte sedimentation rate [ESR] 37 [IQR 26–57] vs 27 [IQR 12–41], *p* <0.001), reflecting more severe disease at diagnosis than patients who did not commence early anti-TNF [Table 1; Supplementary Table 2]. Additionally, compared with their counterparts, patients with early anti-TNF were more likely to have an ileocolonic disease distribution [71% vs 45%, *p* <0.001]. As expected, most POPOs were more frequently present in patients receiving early anti-TNF, with 86% of patients treated with early anti-TNF having at least one POPO compared with 59% in the other group [*p* <0.001] [Supplementary Table 3]. In patients with moderate-to-severe disease activity at diagnosis, 76% had at least one POPO, whereas in patients with mild or inactive disease at diagnosis, this was 60%.

### 3.2. Outcomes at 1 year

**Figure 1:**
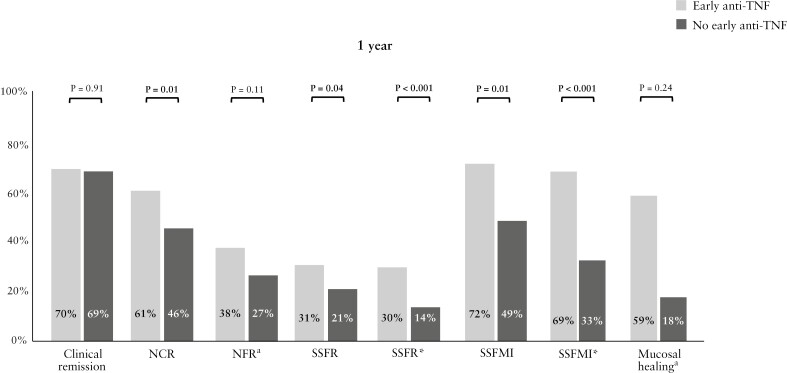
Treatment targets at 1 year in children with Crohn’s disease, stratified by early anti-TNF use. ^a^These outcomes were only evaluated in patients with available faecal calprotectin [FCP] results. ^b^Mucosal healing was estimated by the Mucosal Inflammation Noninvasive [MINI] Index. CRP, C-reactive protein; NCR, normal CRP remission [remission with CRP <0.5 mg/dl]; NFR, normal FCP remission [remission with faecal calprotectin <250 μg/g]; SSFR, sustained steroid-free remission; SSFR*, SSFR without treatment intensification; SSFMI, sustained steroid-free mild or inactive disease; SSFMI*, SSFMI without treatment intensification.

At 1 year, 21% of patients achieved SSFR* [Table 2]. Whereas 64% of patients were in SFR at 1 year, only 25% of patients were in SSFR, of whom 13 [4%] had required treatment intensification [one escalated to vedolizumab and one required luminal surgery within 365 days in the early anti-TNF cohort; 11 escalated to anti-TNF [six infliximab, five adalimumab] after 90 days in the no early anti-TNF cohort]. In total, 11 patients required luminal resection within the first year [four in the early anti-TNF group, seven in the no early anti-TNF group]. Compared with their counterparts, patients with early anti-TNF had higher rates of SSFR* (39/131 [30%] vs 26/185 [14%], *p* <0.001), as well as higher SSFMI* (117/130 [90%] vs 154/185 [83%], *p* <0.001), and NCR (74/121 [61%] vs 75/162 [46%], *p* = 0.001) at 1 year, but not higher clinical remission rates [70% vs 69%, *p* = 0.91] [Figure 1, Table 2].

**Table 2:** Effect of early therapy on treatment targets at 1 year in children with Crohn’s disease.

Outcome at 1 year, [%]	Early anti-TNF therapy [*n* = 135; 41%]	No early anti-TNF therapy [*n* = 196; 59%]	Total [*n* = 331]	*p*-value
Remission	90/129 [70]	128/185 [69]	218/314 [69]	0.91
NCR	74/121 [61]	75/162 [46]	149/283 [53]	**0.01**
NFR	29/76 [38]	27/101 [27]	56/177 [32]	0.11
SFR	85/130 [65]	116/185 [63]	201/315 [64]	0.63
SSFR	40/131 [31]	38/185 [21]	78/316 [25]	**0.04**
SSFR*	39/131 [30]	26/185 [14]	65/316 [21]	**<0.001**
SSFR* from 6 months^a^	50/129 [39]	38/183 [21]	88/312 [28]	**<0.001**
SFMI	117/130 [90]	154/185 [83]	271/315 [86]	0.09
SSFMI	92/127 [72]	86/177 [49]	178/304 [59]	**<0.001**
SSFMI*	88/127 [69]	58/177 [33]	146/304 [48]	**<0.001**
SSFMI* from 6 months^b^	98/127 [77]	66/179 [37]	164/306 [54]	**<0.001**
Endoscopic inflammation as estimated by MINI-index				0.50
Mucosal healing	47/71 [66]	42/74 [57]	89/145 [61]	
Mild inflammation	13/71 [18]	17/74 [23]	30/145 [21]	
Moderate/severe inflammation	11/71 [16]	15/74 [20]	26/145 [18]	

Varying denominators are the result of different starting points for certain outcomes, the underlying criteria for outcomes and missing data.

TNF, tumour necrosis factor; CRP, C-reactive protein; FC, faecal calprotectin; NCR, normal CRP remission [remission with CRP <0.5 mg/dl]; NFR, normal FCP remission [remission with faecal calprotectin <250 μg/g]; SFR, steroid-free remission; SSFR, sustained steroid-free remission; SSFR*, SSFR without treatment intensification, SFMI, steroid-free mild or inactive disease; SSFMI, sustained SFMI; SSFMI*, SSFMI without treatment intensification; MINI-index, mucosal inflammation non-invasive index.

^a^Defined as being continuous steroid-free remission without treatment intensification from 6 months after diagnosis up to the time-point of assessment.

^b^Defined as being in continuous steroid-free mild or inactive disease without treatment intensification from 6 months after diagnosis up to the time point of assessment.

The MINI-index at 1 year could be determined for 145 [44%] patients [Table 2]. Comparing patients treated with early anti-TNF with those without early anti-TNF, there were no significant differences observed in the median MINI-index [2.8 vs 5.6, *p* = 0.052] or in the proportion of patients with MINI-index <3 as a proxy for MH [66% vs 57%, *p* = 0.24] [Figure 1].

#### 3.2.1. Association between early anti-TNF and outcomes at 1 year

The adjusted OR for the association between early anti-TNF and the probability of achieving SSFR*, after controlling for baseline covariates in our regression model, was 3.09 [95% CI 1.69-5.67] [Supplementary Table 4].

Based on the standardisation model, the crude RR of achieving SSFR* at 1 year in the early anti-TNF group compared with the no early anti-TNF group was 2.95 [95% CI 1.63-5.36], indicating a significantly increased benefit of early anti-TNF among our patients [*p* <0.001] [Table 3]. This effect was even larger for achieving SSFMI* with a relative risk of 4.67 [95% CI 2.46-8.87, *p* <0.001]. For NCR and MH as estimated by MINI-index, the effect of early anti-TNF did not reach statistical significance.

**Table 3:** Crude odds ratio and risk ratio of early anti-TNF therapy for 1-year outcomes in pediatric Crohn’s disease patients.

Outcome	OR^a^	95% CI	*p*-value	RR^a^	95% CI	*p*-value
SSFR*	3.25	1.76-6.00	**<0.001**	2.95	1.63-5.36	**<0.001**
SSFR	1.82	1.05-3.14	**0.03**	1.65	1.00-2.72	**0.049**
SSFMI*	6.32	3.37-11.85	**<0.001**	4.67	2.46-8.87	**<0.001**
NCR	1.65	0.94-2.88	0.08	1.34	0.91-1.97	0.14
Mucosal healing [as estimated by MINI index]	1.64	0.75-3.57	0.22	1.28	0.81-2.01	0.29

CRP, C-reactive protein; TNF, tumour necrosis factor; OR, odds ratio; CI, confidence interval; RR, relative risk; NCR, normal CRP remission [remission with CRP <0.5 mg/dl]; SSFR[*], sustained steroid-free remission [*without treatment intensification]; SSFMI[*], sustained steroid-free mild or inactive disease [*without treatment intensification]; MINI-index, mucosal inflammation non-invasive index.

^a^Ratios represent the comparison of early anti-TNF therapy against no early anti-TNF therapy.

#### 3.2.2. Association between early anti-TNF and outcomes at 1 year in subgroups of patients

After stratifying the patient population by baseline disease activity, we found that the RR for achieving SSFMI* with early anti-TNF compared with no early anti-TNF was much higher, and statistically significant, in patients with moderate-to-severe disease activity at diagnosis (RR 5.50 [95% CI 2.51-12.05], *p* <0.001) than in patients with mild or inactive disease activity at diagnosis (RR 2.91 [95% CI 0.92-9.11], *p* = 0.07), regardless whether or not POPOs were present [Table 4]. Similarly, the RR for early anti-TNF was much higher in patients with any POPO (RR 5.05 [95% CI 2.45-10.43]) than in patients without any POPO (RR 3.41 [95% CI 0.54-21.7]). The effect of early anti-TNF for achieving SSFMI* adjusted for other baseline covariates stratified by patient subgroup is demonstrated in Supplementary Table 5.

**Table 4:** Crude odds ratio and risk ratio of early anti-TNF therapy for sustained steroid-free mild or inactive disease without treatment intensification at one year by paediatric Crohn’s disease risk groups.

Subgroup	OR	95% CI	*p*-value	RR	95% CI	*p*-value
Mild/inactive disease at diagnosis	3.86	1.27–11.72	**0.02**	2.91	0.92–9.11	0.07
Moderate/severe disease at diagnosis	7.22	3.45–15.13	**<0.001**	5.50	2.51–12.05	**<0.001**
No POPO	4.63	1.03–20.87	**0.046**	3.41	0.54–21.7	0.19
Any POPO	6.66	3.48–12.72	**<0.001**	5.05	2.45–10.43	**<0.001**

TNF, tumour necrosis factor; OR, odds ratio; CI, confidence interval; RR, relative risk; POPO, predictor of poor outcome.

#### 3.2.3. Growth

In Table 5, growth parameters of the study population at diagnosis and at 1 and 2 years after diagnosis, stratified by early therapy, are demonstrated. At diagnosis, mean BMI Z-score for all patients was significantly lower than population mean for BMI [-0.96; *p* <0.001]. Mean HFA Z-score at baseline was slightly reduced, but this was not significant [-0.09; *p* = 0.16]. Patients with early anti-TNF had lower baseline BMI Z-scores than patients without early anti-TNF [-1.21 vs -0.79, *p* = 0.01] By 1 year, linear growth patterns had not significantly changed [ΔHFA Z-score + 0.03; *p* = 0.33], but BMI had significantly improved [ΔBMI Z-score + 1.03, *p* <0.001]. When stratified by therapy group, patients with early anti-TNF had significantly better improvement of ΔBMI Z-score [+1.18 and + 0.88, respectively; *p* = 0.02], but not better improvement ΔHFA Z-scores. Stratification by risk group based on disease activity at diagnosis did not show a more beneficial effect of early anti-TNF on growth parameters at 1 or 2 years in patients with moderate-severe disease as compared with mild-inactive disease [data not shown].

**Table 5:** Effect of early therapy on growth outcomes in children with Crohn’s disease.

Growth parameter	Early anti-TNF [*n* = 135; 41%]	No early anti-TNF [*n* = 196; 59%]	Total [*n* = 331]	*p*-value^a^
HFA Z-score at diagnosis, mean [SD]	130/135 -0.06 [1.10]	190/196 -0.11 [1.31]	320/331 -0.09 [1.19]	0.7
BMI Z-score at diagnosis, mean [SD]	129/135 -1.21 [1.48]	190/196 -0.79 [1.50]	319/331 -0.96 [1.51]	**0.01**
ΔHFA Z-score by 1 year, mean [SD]^b^	104/135 +0.05 [0.39]; *p* = 0.16	145/196 +0.01 [0.59]; *p* = 0.80	249/331 +0.03 [0.50]; *p* = 0.33	0.56
ΔHFA Z-score by 2 years, mean [SD]^b^	37/72 +0.15 [0.62] *p* = 0.09	83/121 +0.20 [0.60] ***p* = 0.009**	120/192 +0.18 [0.60]; ***p* = 0.002**	0.65
ΔBMI Z-score by 1 year, mean [SD]^b^	100/135 +1.18 [1.05]; ***p* <0.001**	142/196 +0.88 [1.07]; ***p* <0.001**	242/331 +1.03 [1.07]; ***p* <0.001**	**0.02**
ΔBMI Z-score by 2 years, mean [SD]^b^	35/71 +1.41 [1.30]; ***p* <0.001**	81/121 +1.05 [1.07]; ***p* <0.001**	116/192 +1.21 [1.19]; ***p* <0.001**	0.11

scores were obtained by standardising growth parameters using the WHO Growth Reference standards.

TNF, tumour necrosis factor; SD, standard deviation; HFA, height for age; BMI, body mass index.

^a^Independent two-sample t test.

^b^Paired sample t test.

#### 3.2.4. Health related quality of life

HRQOL significantly improved after 1 year of therapy [mean ΔIMPACT-III score + 11.2; *p* <0.001] [Supplementary Table 6]. Stratified by early anti-TNF use, no differences were observed at 1 and 2 years for the overall mean ΔIMPACT-III scores [*p* = 0.2], or the ΔIMPACT-III scores for any of the six subdomains of the questionnaire [data not shown]. The improvement in EQ-5D-5L VAS score at 1 year was significantly higher for patients on early anti-TNF [ΔVAS + 17.4 vs +9.8; *p* = 0.03]. No significant differences were observed when stratified by risk based on disease activity at diagnosis [data not shown].

### 3.3. Outcomes at 2 years or last follow-up

**Figure 2:**
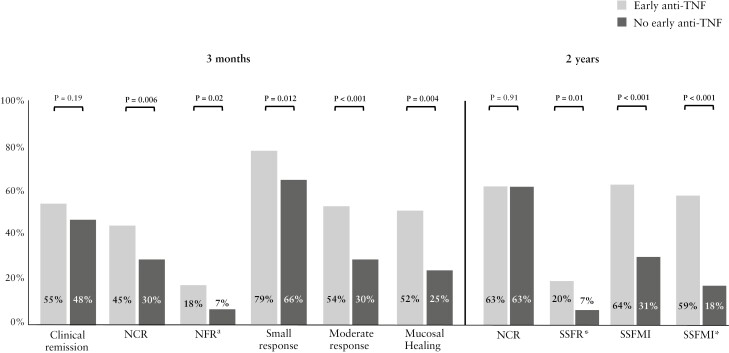
Treatment targets at 3 months and 2 years in children with Crohn’s disease, stratified by early anti-TNF use. ^a^These outcomes were only evaluated in patients with available faecal calprotectin results. ^b^Mucosal healing was estimated by the Mucosal Inflammation Noninvasive [MINI] Index. TNF, tumour necrosis factor; C-reactive protein; FCP, faecal calprotectin; NCR, normal CRP remission [remission with CRP <0.5 mg/dl]; NFR, normal FCP remission [remission with faecal calprotectin <250 μg/g]; SSFR*, sustained steroid-free remission without treatment intensification; SSFMI, sustained steroid-free mild or inactive disease; SSFMI*, SSFMI without treatment intensification.

In total, 192/331 patients (58%; 121/192 [63%] no early anti-TNF) had at least 2 years of follow-up, of whom 21/180 [11%] achieved SSFR* at 2 years and 55/167 [33%] achieved SSFMI* at 2 years [Supplementary Table 7; varying denominators result from different initial values and starting points for each outcome and missing data at subsequent visits]. Patients with early anti-TNF had higher rates of SSFR* [20% vs 7%; *p* = 0.011] and SSFMI* [59% vs 18%; *p* <0.001] than patients without early anti-TNF [Figure 2].

The median duration until last follow-up was 718 days [IQR 490–1004]. The follow-up duration was significantly higher in the no early anti-TNF group (728 days [IQR 559–1079]) than in the anti-TNF group (703 days [IQR 415–832]; *p* = 0.008) but this difference was clinically irrelevant Up to the last visit, 17 [5%] patients required luminal resection (six [4.4%] in the early anti-TNF group, 11 [5.6%] in the no early anti-TNF group; *p* = 0.6), after a median duration of 248 days [IQR 56–462] since diagnosis. Only six patients required surgery after the first year. Fifteen patients had treatment escalation to a second-line biologic agent up to the last follow-up (eight [6%] in early anti-TNF group, seven [4%] in the no early anti-TNF group; *p* = 0.3), after a median of 559 days [IQR 128–308], which was significantly shorter for patients with no early anti-TNF [316 days [IQR 76 - 166] vs 836 days [IQR 224 - 398], log-rank test *p* = 0.026].

### 3.4. Outcomes at 3 months

Patients with early anti-TNF were not more likely to be in clinical remission at 3 months [55% vs 48%, *p* = 0.19][Figure 2; Supplementary Table 8]. However, patients with early anti-TNF demonstrated a more profound reduction in disease activity, as reflected by higher rates of small and moderate response to treatment [79% vs 66%, *p* = 0.012 and 54% vs 30%, respectively; *p* <0.001] and NCR [45% vs 30%, *p* = 0.006].

### 3.5. Sustaining remission by early therapy

The probability of being in SSFR* for those patients that were in SFR at 3 months was 46%, which was significantly higher for patients with early anti-TNF [59%] than for patients without early anti-TNF [35%; *p* = 0.004] [Supplementary Table 9]. Out of all patients that were in SFMI at 3 months, 88/105 [84%] reached SSFMI* at 1 year with early anti-TNF and 58/127 [46%] without early anti-TNF [*p* <0.001].

## 4. Discussion

This is the first predominantly European, multinational, real-world data study on outcomes of early anti-TNF in children with CD. The findings of this study confirm the hypothesis that early anti-TNF in this population leads to improved clinical outcomes at 1 year, including a nearly 3-fold higher chance of SSFR*. These findings are in line with previous studies.

According to a systematic review and meta-analysis on the efficacy of early biologic treatment in CD by Ungaro *et al.*, the pooled OR based on three studies for clinical remission at 1 year in paediatric patients given early anti-TNF was 3.07 [95% CI 1.59-5.94], similar to our SSFR* RR of 2.95 [95% CI 1.63-5.36].^5,22–24^ The North American observational RISK cohort study found that early treatment with anti-TNF was superior to early treatment with [and without] immunomodulatory therapy in achieving steroid- and surgery-free remission [SFR] in children with new-onset CD, with slightly lower RRs [1.41 and 1.57, respectively].^5^ The differences in the definition of the selected primary outcome and the difference in statistical analyses [propensity score matching in their study, regression standardisation in ours] could explain the observed differences. Also, our study was mainly conducted in European centres, which adhere to the ESPGHAN guideline advocating combination therapy of [early] anti-TNF with an immunomodulator to reduce immunogenicity. The use of combination therapy in the majority of patients in our study [72%] may have contributed to more pronounced effects in terms of achieving SSFR*.

Anti-TNF has been shown to contribute to restoration of linear growth in children.^5,25–29^ We did not observe a significant difference in the increase of linear growth at 1 or 2 years by early therapy use. The disparity between this and the cohort of Walters *et al*., where growth was reported to improve, may be attributed to the baseline HFA Z-scores being lower in their study than ours [HFA Z-score -0.30 vs -0.09].^5^

It has been debated whether early anti-TNF can alter the natural history of CD. In a nationwide study using health administrative data from Israel, Focht *et al.* found that early initiation of anti-TNF [<3 months] did decrease the likelihood of IBD-related surgery in children and adults with CD, but only when compared with late [2–3 years] initiation of anti-TNF (survival probabilities:0–3 months 0.75 [95% CI 0.71-0.79] vs 2–3 years 0.70 [95% CI 0.66–0.73]; *p* <0.05).^30^ Three other large cohort studies in children evaluated the effectiveness of early anti-TNF on disease progression, and consistently demonstrated that anti-TNF, started within 3 months after diagnosis, delayed CD progression to B2 disease, B3 disease, or both.^4,6,31^ The number of patients who developed complicated disease after baseline up to the last follow-up was only one in our study, and the number of patients requiring bowel resection up to the last follow-up was only 17. This could be because of the relatively short follow-up of our study (median length of follow-up 718 days [IQR 490–1004 days]), but could also reflect the consequences of improved treatment strategies and timely diagnosis, leading to less bowel wall damage. When focusing on short- and intermediate-term outcomes, our data demonstrate improved rates of achieving clinical and biochemical remission with early anti-TNF.

A big strength of choosing time-based outcomes, such as the SSFR[*] and SSFMI[*] outcomes, is their ability to depict disease activity status over an extended duration, in contrast to outcomes lacking a time element such as [biochemical] remission, which only reflect the disease status at a specific time point. However, the major challenge in selecting time-based outcomes is the inherent bias introduced when the comparison naturally includes an element of time. This bias emerges due to the differing trajectories of patients who receive early anti-TNF treatment vs those who do not, with the latter following a course which consumes time, causing a natural delay in reaching their milestones. In an effort to eliminate this time-related bias, we balanced both groups by comparing rates of SSFR* and SSFMI* after attaining SFR or SFMI at the 3-month mark, to determine if maintaining remission for 9 months was equivalent between both groups. These analyses showed superior effectiveness of sustaining controlled disease with early anti-TNF therapy.

Our study is the first to evaluate the effect of early anti-TNF on patient wellbeing, showing a significant and more substantial improvement in HRQOL at 1 year, as assessed by the ΔEQ-5D-5L VAS, in patients with early anti-TNF compared with patients without early anti-TNF [+17.4 vs +9.8; *p* = 0.03].

It is unclear if early anti-TNF affects the chance of achieving MH, which is an important treatment target. Endoscopic reassessment in patients with good clinical outcomes is rarely performed, as endoscopies are invasive procedures. Rates of MH as estimated by MINI-index were similar between early anti-TNF vs no early anti-TNF, but could only be evaluated in a minority of patients because of many missing values for FCP results. Contrarily, Ungaro *et al*. showed that early biologic treatment in IBD patients resulted in higher MH rates (pooled OR 2.37 [95% CI 1.78-3.16]).^22^ However, the cut-off for early vs late initiation and definitions of MH varied greatly between the studies. Two small prospective paediatric studies evaluating early [<1 year] vs late [>1 year] anti-TNF did not demonstrate any significant differences in MH rates (45% vs 32%; OR 1.73 [95% CI 0.45-6.63] and 65% vs 13%, OR 1.15 [95% CI 0.26-5.11]), but this could be due to small sample sizes [*n* = 37 and *n* = 30, respectively].^32,33^

This study has demonstrated that early anti-TNF is particularly beneficial for patients with a high-risk of disabling disease, as characterised by the presence of POPOs, with an RR of 5.05 [95% CI 2.45-10.43] for SSFMI*. Many of these POPOs lack adequate criteria or descriptions and rely on subjective assessment.^8^ Furthermore, most POPOs have solely been studied in the adult IBD population. In our study, using well-defined criteria for each POPO, we observed that the risk for achieving SSFMI* did not reach statistical significance in patients without any POPO, supporting a step-up treatment approach [and no early use of anti-TNF] for this patient population. Furthermore, our study revealed that patients with moderate-to-severe disease activity at diagnosis had a nearly 2-fold higher RR for achieving SSFMI* with early anti-TNF compared with patients with mild or inactive disease at diagnosis (RR 5.50 [95% CI 2.51-12.05] vs 2.91 [0.92-9.11]). This is consistent with the TISKids study, a randomised controlled trial [RCT] comparing upfront infliximab [5 infusions] with exclusive enteral nutrition or steroids in treatment-naïve paediatric patients with moderate-to-severe luminal CD, showing that a significantly greater proportion of children with CD treated with infliximab achieved short-term clinical and endoscopic remission [Week 10], as well as clinical remission without treatment intensification at 1 year.^34^

### 4.1. Strengths and limitations

This study has several strengths. First, the prospective study design and detailed and standardised nature of the clinical data collection enhanced the reliability and accuracy of the findings. We used robust data validation procedures, further ensuring the integrity of the collected data. Whereas population-based studies using health administrative databases may have the advantage of an even larger sample size, they often lack clinical details regarding disease phenotype and markers of endoscopic, laboratory, or clinical severity. We included a large number of patients, over 300 in total, from multiple international centres. The real-world setting, characterised by the heterogeneity of the included population, strengthens both the representativeness and external validity of this study. Although the majority of patients were recruited from centres in the UK and The Netherlands, all participating centres adhered the same ESPGHAN guideline, thereby enhancing the generalisability of the study results. We evaluated various endpoints, allowing for a comprehensive assessment of the effects of early anti-TNF. Last, we have been able to analyse the treatment targets growth and quality of life, two important but often understudied outcomes in paediatric IBD studies.

There are some limitations. First, this was an observational study with very broad entry criteria and clinical decisions made by the team looking after them and not as part of a protocol. Thus, a wide range of IBD patients with various demographics and clinical characteristics were recruited, which provides better representation of the average IBD patient to study therapy effectiveness in real world clinical practice settings. Other unrecorded factors, such as patient preferences, distance to the hospital, centres’ access to anti-TNF, costs and health insurance policies, could potentially influence a physician’s decision to initiate early anti-TNF. However, in this large cohort group from predominantly specialised PIBD centres, the primary drivers for such a decision would typically be disease severity and phenotype. We applied the method of standardisation to emulate an RCT, which is valid under the assumption of no unmeasured confounders. Using penalised regression modelling allowed us to incorporate a large number of confounding factors, such as the POPOs and other baseline covariates. Although RCT’s are regarded as the gold standard for therapy efficacy, patients enrolled in RCTs are often a highly selected population that may differ from the target population because of restricted eligibility criteria.^35^

Routine assessment of biochemical markers such as FCP was not part of the observational study protocol, resulting in a considerable number of missing values for this parameter. As a consequence of missing data, outcomes that incorporate FCP, such as the MINI-index, could only be assessed in a limited number of patients, and these results should be interpreted with caution. Also, the absence of data could introduce bias, since patients with available FCP results might have more poorly controlled disease. Consequently, baseline FCP could not be incorporated as confounding factor in the prediction model. However, it was used for imputing other essential variables, including CRP, which were included in the model. This was done because excluding cases with missing data, ie, full case analysis, could have introduced bias, as the missingness could be related to other variables.

### 4.2. Implications and future research

The outcomes of this study present compelling evidence for the revision of the current ESPGHAN CD guidelines, which advocates a risk-stratified strategy, recommending the initiation of upfront or early anti-TNF in patients with one or more POPOs. Our study has demonstrated that although patients with any POPO indeed have better outcomes with early anti-TNF, patients with moderate-to-severe disease at diagnosis also benefit from early anti-TNF therapy, regardless of the presence of any POPO. As a result, there is a need to broaden the scope of indications for early anti-TNF. On the other hand, patients without any POPOs, as well as those with no or mild disease, exhibited lower RRs that did not reach statistical significance. For these low-risk patients, a step-up approach may suffice.

Ultimately, the goal is to develop a clinical decision tool that combines multi-omics data and clinical data to predict disease outcomes, which could be used to stratify and personalise treatment. A consensus has been established on some key prognostic clinical factors in paediatric IBD, but this is not yet the case for multi-omics biomarkers. Recent work has suggested the potential role of serological, gut microbial, metabolomic, genetic [genome-wide association studies or HLA-DQA1 phenotyping], and proteomic profiles to guide early stratification.^36,37^

### 4.3. Conclusion

Despite commendable efforts to stratify patients based on multi-omics data at diagnosis to select early therapy, such decision tools are currently lacking. In the absence of these tools, risk stratification for early therapy remains symptom- and target-driven. This study provides evidence to support the use of early anti-TNF therapy in paediatric patients with newly diagnosed Crohn’s disease, in particular in presumed high-risk patients, ie, those with moderate-to severe disease activity or the presence of any POPO.

## Supplementary Material

jjad197_suppl_Supplementary_Data

jjad197_suppl_Supplementary_Tables_S1-S9

## Data Availability

De-identified participant data that underlie the results reported in this article [text, tables, figures, and appendices] will be made available upon reasonable request. Additional documents [study protocol, informed consent form, statistical analysis plan, and analytical code] will also be available upon reasonable request. Data will be made available beginning 9 months and ending 36 months following article publication, to researchers who provide a methodologically sound proposal, and to achieve aims in the approved proposal, as determined by the corresponding author of this study. Proposals may be submitted up to 36 months following article publication and should be directed to the corresponding author. Patients and/or the public were not involved in the design, conduct, reporting, or dissemination plans of this research.
