# Supplementary material for: Improved Clinical Outcomes With Early Anti-Tumour Necrosis Factor Alpha Therapy in Children With Newly Diagnosed Crohn’s Disease: Real-world Data from the International Prospective PIBD-SETQuality Inception Cohort Study
Source: J Crohns Colitis. 2023 Nov 27;18(5):738–50. doi: 10.1093/ecco-jcc/jjad197 (PMC11140629; doi:10.1093/ecco-jcc/jjad197)
Supplement: jjad197_suppl_Supplementary_Data [file jjad197_suppl_supplementary_data.docx]

**SUPPLEMENTAL METHODS**

**Data collection**

*Laboratory measurements*

Laboratory tests measured including serum albumin level, haemoglobin level, platelet count, leukocyte count, C-reactive protein (CRP) level, erythrocyte sedimentation rate (ESR) and faecal calprotectin (FCP). Results of blood and stool samples were included if obtained within a specified visit time window. For blood samples, this was 15 before to 7 days after the study visit date. For stool samples, this window was dependent on the visit (baseline: -120 to +7 days; visit 2: -21 to +7 days; visit 3: -30 to +7 days; visit 4 and further: -60 to +7 days).

*Therapy*

Therapy groups included 5-aminosalicylates (5-ASA; oral and rectal), immunomodulators (azathioprine, 6-mercaptopurine, thioguanin, methotrexate), EEN or other exclusion diet, systemic steroids (oral or rectal), and biologics. IFX and ADA were considered first-line biologics, ustekinumab and vedolizumab second-line biologics. Patients were initiated on anti-TNF agents (originators or biosimilars) at the physician’s discretion on standard doses according to paediatric CD guidelines, and could receive other concomitant treatments (immunomodulators, steroids, 5-ASA, EEN).

*Paris classification*

Baseline disease location, behavior, and perianal disease were scored according to the Paris classification, based on findings at endoscopy and imaging performed within 90 days after diagnosis. If endoscopy or small bowel imaging was performed but specific findings (e.g. narrowing) were unreported/unmentioned, we assumed results to be normal. Stricturing disease (B2) was defined as a SES-CD subscore for narrowing ≥ 1 at ileocolonoscopy or constant luminal narrowing in combination with prestenotic dilatation as observed by imaging. Internal penetrating (B3) disease was defined as a bowel perforation, intra-abdominal fistulas, inflammatory masses and/or abscesses (excluding the vagina or perianal region) observed by imaging. Uncomplicated disease was referred to as inflammatory (B1) disease. If no imaging was performed, patients were classified as B1. Perianal disease was defined as fistulas (indolent or active), anal canal ulcers or abscesses in the perianal or perirectal region, observed on physicial examination or by MRI imaging. Skin tags or fissures were not considered perianal disease.

*MINI-index*

To evaluate mucosal healing, we used the Mucosal Inflammation Noninvasive Index (MINI index), a non-invasive tool to assess mucosal inflammation in children with CD.(14) This score is based on the stooling item of the wPCDAI, CRP, ESR and level of FCP and can identify patients with mucosal healing with high sensitivity and specificity. A score <8 reflects mucosal healing (equivalent to a SES-CD <3), a score of 8-11 reflects mild inflammation (equivalent to a SES-CD 3-9), while a score > 11 reflects moderate-to-severe inflammation (equivalent to a SES-CD ≥10).

*Quality of life assessment*

The IMPACT-III questionnaire is a validated 35-item IBD-specific measure of HRQOL for children. It encompasses 6 domains: IBD symptoms, systemic symptoms, emotional functioning, social functioning, body image, and treatment/intervantions. The IMPACT-III uses a 5-point Likert scale for all items. Scores range from 35 to 175 and, as per author’s guidelines, scores are then linearly and reversely transformed to a range of 0–100, with 0 indicating the lowest HRQoL and 100 indicating the highest HRQoL.(17). Missing data on items of the IMPACTIII questionnaire were treated as indicated in the IMPACT-III User’s Guide, total and domain scores were computed as the mean of all completed items. The EQ-5D-5L questionnaire is a generic HRQOL instrument composed of two parts: a descriptive part consisting of five dimensions of health (mobility, self-care, usual activities, pain/discomfort, and anxiety/depression) and the visual analogue scale (VAS).(18) EQ-5D-5L questionanires were completed by the patient (EQ-5D-Youth) and/or one of both parents (EQ-5D-Youth-Proxy). The EQ-5D-Youth-Proxy VAS was used if the EQ-5D-Youth VAS was missing, as these scores showed fairly good correlation (Pearson’s R 0.74 [95%CI 0.72 – 0.76]).
