# Supplementary material for: Improved Clinical Outcomes With Early Anti-Tumour Necrosis Factor Alpha Therapy in Children With Newly Diagnosed Crohn’s Disease: Real-world Data from the International Prospective PIBD-SETQuality Inception Cohort Study
Source: J Crohns Colitis. 2023 Nov 27;18(5):738–50. doi: 10.1093/ecco-jcc/jjad197 (PMC11140629; doi:10.1093/ecco-jcc/jjad197)
Supplement: jjad197_suppl_Supplementary_Tables_S1-S9 [file jjad197_suppl_supplementary_tables_s1-s9.docx]

**Supplemental Table 1 – Paediatric Crohn’s disease patients recruited per participating center of the PIBD-SETQuality study, eligible for analysis**

| **Country** | **Center** | **Number of recruited patients (%)** |
| --- | --- | --- |
| **Germany** | Dr von Hauner Children’s Hospital, Munich | 7 (2.1) |
| **France** | Centre Hospitalier Universitaire Descarte, Necker–Enfants Malades, Paris | 1 (0.3) |
| **Israel** | Wolfson Medical Center, Holon | 5 (1.5) |
| **Israel** | Shaare Zedek Medical Center, Jerusalem | 10 (3.0) |
| **Israel** | Schneider Children’s Medical Center of Israel, Petach Tikva | 2 (0.6) |
| **Italy** | Sapienza University Hospital, Rome | 5 (1.5) |
| **Italy** | Federico II University Hospital, Naples | 4 (1.2) |
| **Japan** | National Center for Child Health and Development, Tokyo | 11 (3.3) |
| **Malaysia** | University of Malaya Medical Centre, Kuala Lumpur | 4 (1.2) |
| **The Netherlands** | Erasmus Medical Center – Sophia Children’s Hospital, Rotterdam | 70 (21.2) |
| **The Netherlands** | Medisch Spectrum Twente, Enschede | 9 (2.7) |
| **The Netherlands** | Rijnstate Ziekenhuis, Arnhem | 4 (1.2) |
| **The Netherlands** | Jeroen Bosch Ziekenhuis, Den Bosch | 3 (0.9) |
| **The Netherlands** | Amsterdam University Medical Center, Amsterdam | 13 (3.9) |
| **Serbia** | University Children’s Hospital, Belgrade | 12 (3.6) |
| **United Arab Emirates** | Al Jalila Children’s Speciality Hospital, Dubai | 1 (0.3) |
| **United Kingdom** | Royal London Hospital, London | 57 (17.2) |
| **United Kingdom** | Alder Hey Children’s Hospital, Liverpool | 23 (7.0) |
| **United Kingdom** | Royal Hospital for Sick Children, Edinburgh | 10 (3.0) |
| **United Kingdom** | Southampton Children’s Hospital, Southampton | 6 (1.8) |
| **United Kingdom** | Royal Hospital for Children Glasgow, Glasgow | 13 (3.9) |
| **United Kingdom** | Birmingham Women’s and Children’s Hospital, Birmingham | 13 (3.9) |
| **United Kingdom** | Sheffield Children’s Hospital, Sheffield | 9 (2.7) |
| **United Kingdom** | Oxford Children’s Hospital, Oxford | 20 (6.0) |
| **United Kingdom** | Royal Free Hospital, London | 4 (1.2) |
| **United Kingdom** | Nottingham Children’s Hospital, Nottingham | 5 (1.5) |
| **United Kingdom** | Royal Devon and Exeter Hospital, Exeter | 5 (1.5) |
| **United Kingdom** | University College Hospital, London | 4 (1.2) |
| **United Kingdom** | King’s College, London | 1 (0.3) |
| **Total** |  | **331** |

**Supplemental Table 2 – Laboratory results at diagnosis of the paediatric Crohn’s disease population, stratified by early anti-TNF use**

| **Laboratory result** | **Early anti-TNF therapy**  **(n = 135; 59%)** | **No early anti-TNF therapy**  **(n = 196; 41%)** | **Total  (n = 331)** | **p-value** |
| --- | --- | --- | --- | --- |
| Haemoglobin (mmol/l), mean (SD)  < 6 mmol/l, n (%) | 6.9 (1.0)  21 (16) | 7.1 (1.0)  22 (12) | 7.0 (1.0)  43 (14) | **0.049**  0.33 |
| C-reactive protein (mg/l), median (IQR)  > 5 mg/l, n (%) | 25 (13 – 51)  114 (90) | 14 (4 – 40)  117 (70) | 18 (7 – 47)  231 (79) | **<0.001**  **<0.001** |
| Albumin (g/l), mean (SD)  < 35 g/l, n (%) | 34 (7)  74 (56) | 36 (7)  72 (38) | 35 (7)  146 (46) | **0.001**  **0.002** |
| Platelet count (10^9^/l), median (IQR) | 448 (358 - 549) | 419 (329 – 508) | 430 (347 - 521) | 0.06 |
| Leukocyte count (10^9^/l), median (IQR) | 9.3 (7.1 – 11.7) | 8.4 (7.2 – 10.6) | 8.6 (7.1 – 10.9) | 0.10 |
| Erythrocyte sedimentation rate, median (IQR) | 37 (26 – 57) | 27 (12 – 41) | 32 (17 – 48) | **<0.001** |
| Faecal calprotectin (mcg/g), median (IQR)  > 250 mcg/g, n (%) | 1600 (992 – 2450)  67 (92) | 992 (566 – 2590)  77 (90) | 1430 (686 – 2553)  114 (91) | 0.08  0.83 |

Missing values for each laboratory result: CRP 37; albumin 11; haemoglobin 18; platelet count 20; leukocyte count 25; ESR 48; faecal calprotectin 172.

**Supplemental Table 3 – Predictors of poor outcome in the paediatric Crohn’s disease population, stratified by early anti-TNF use**

| **Predictor of poor outcome (POPO)** | **Early anti-TNF therapy**  **(n = 135; 59%)** | **No early anti-TNF therapy**  **(n = 196; 41%)** | **Total  (n = 331)** | **p-value** |
| --- | --- | --- | --- | --- |
| POPO deep colonic ulcer^a^ | 53 (42) | 48 (27) | 101 (33) | **0.009** |
| POPO extensive disease^b^ | 58 (43) | 22 (27) | 110 (34) | **0.005** |
| POPO growth delay^c^  (HFA Z-score < -1.5) | 21 (16) | 23 (12) | 44 (14) | 0.40 |
| POPO complicated disease^d^ | 31 (23) | 27 (14) | 58 (18) | **0.046** |
| POPO perianal disease^e^ | 46 (34) | 19 (10) | 65 (20) | **<0.001** |
| Any (1 or more) POPO^f^ | 117 (86) | 116 (59) | 233 (70) | **<0.001** |

^a^ Defined as a subscore of ≥2 on the ‘ulcer’ item of the SES-CD (large or very large ulcers)

^b^ Defined as ileocolonic disease and proximal disease (L3 + L4a and/or L4b as per Paris classification)

^c^ Defined as a height-for-age Z-score < -1.5

^d^ Defined as stricturing (B2) and/or penetrating (B3) disease, or inflammatory (B1) disease with narrowing without prestenotic dilatation.

^e^ Defined as fistulas (indolent or active), anal canal ulcers or abscesses in the perianal or perirectal region

Numbers represent n (%).

^f^ Based on the imputed values of all POPOs

Missing values for each POPO: deep colonic ulcer 27; extensive disease 6; growth delay 7; complicated disease 1; perianal disease 0.

**Supplemental Table 4 – Multivariate logistic regression model of predictors for sustained steroid-free remission without treatment intensification in pediatric Crohn’s disease**

| **Predictor** | **Adjusted OR** | **95% CI** | **p-value** |
| --- | --- | --- | --- |
| Early anti-TNF therapy^a^ | **3.09** | **1.69 – 5.67** | <0.001 |
| Age at diagnosis in years | 0.99 | 0.90 – 1.09 | 0.89 |
| Female sex | 0.66 | 0.36 – 1.19 | 0.17 |
| Moderate-to-severe disease activity at diagnosis^b^ | 0.93 | 0.50 – 1.74 | 0.81 |
| CRP level at diagnosis (mg/l) | 1.00 | 0.99 – 1.01 | 0.62 |
| POPO deep colonic ulcer | 0.70 | 0.36 – 1.36 | 0.30 |
| POPO extensive disease | 1.14 | 0.63 – 2.06 | 0.65 |
| POPO complicated disease | 0.65 | 0.30 – 1.40 | 0.28 |
| POPO perianal disease | **0.48** | 0.23 – 1.00 | 0.05 |
| POPO growth delay | 0.66 | 0.28 – 1.57 | 0.35 |

Adjusted odds ratios for predictors of sustained steroid-free remission without treatment intensification utilising a penalised logistic regression model.

Abbreviations: anti-TNF, anti-tumor necrosis factor alpha; CRP, C-reactive protein; POPO, predictor of poor outcome.

^a^Reference group is no early anti-TNF therapy.

^b^Reference group is inactive or mild clinical disease activity.

**Supplemental Table 5 – Multivariate logistic regression models of predictors for sustained steroid-free mild or inactive disease without treatment intensification at one year in pediatric Crohn’s disease**

| **Predictor** | **Mild or inactive disease activity at diagnosis** | | **Moderate or severe disease activity at diagnosis** | | **No predictor of poor outcome^*^** | | **Any predictor of poor outcome^*^** | |
| --- | --- | --- | --- | --- | --- | --- | --- | --- |
|  | **aOR (95% CI)** | **P** | **aOR (95% CI)** | **P** | **aOR (95% CI)** | **P** | **aOR (95% CI)** | **P** |
| Early anti-TNF therapy^a^ | 3.86 (1.35 – 11.00) | **0.02** | 7.22 (3.69 - 14.14) | **<0.001** | 4.67 (1.32 - 16.43) | **0.014** | 6.63 (3.52 - 12.49) | **<0.001** |
| Age at diagnosis, years | 1.13 (0.99 - 1.30) | 0.68 | 0.95 (0.85 - 1.06) | 0.38 | 1.05 (0.91 - 1.20) | 0.52 | 0.99 (0.89 - 1.10) | 0.81 |
| Female sex | 1.00 (0.42 - 2.37) | 0.92 | 1.16 (0.62 - 2.14) | 0.64 | 0.78 (0.33 - 1.83) | 0.57 | 1.25 (0.68 - 2.31) | 0.47 |
| Moderate-to-severe disease activity at diagnosis^b^ | n/a | n/a | n/a | n/a | 0.67 (0.28 - 1.59) | 0.36 | 1.03 (0.52 - 2.05) | 0.86 |
| C-reactive protein level at diagnosis (mg/l) | 0.98 (0.95 - 1.01) | 0.26 | 1.00 (0.99 - 1.00) | 0.83 | 0.98 (0.94 - 1.01) | 0.15 | 1.00 (0.99 - 1.01) | 0.59 |
| POPO deep colonic ulcer | 0.27 (0.07 - 1.00) | **0.046** | 0.72 (0.36 - 1.43) | 0.35 | n/a | n/a | 0.57 (0.29 - 1.12) | 0.10 |
| POPO extensive disease | 0.22 (0.07 - 0.70) | **0.008** | 0.97 (0.51 - 1.84) | 0.91 | n/a | n/a | 0.72 (0.39 - 1.34) | 0.30 |
| POPO complicated disease | 0.56 (0.18 - 1.68) | 0.30 | 0.99 (0.45 - 2.18) | 0.96 | n/a | n/a | 0.81 (0.40 - 1.62) | 0.55 |
| POPO perianal disease | 0.57 (0.19 - 1.73) | 0.32 | 0.95 (0.44 - 2.02) | 0.90 | n/a | n/a | 0.88 (0.45 - 1.70) | 0.70 |
| POPO growth delay | 1.69 (0.49 - 5.90) | 0.41 | 0.64 (0.27 - 1.53) | 0.32 | n/a | n/a | 0.92 (0.44 - 1.93) | 0.82 |

Adjusted odds ratios (aOR) for predictors of sustained steroid-free mild or inactive disease without treatment intensification by subgroup of paediatric Crohn’s disease patients.

*Any of the following POPOs: deep colonic ulcer, extensive disease, growth delay, complicated disease, perianal disease.

^a^Reference group is no early anti-TNF therapy. ^b^Reference group is mild/inactive disease at diagnosis.

**Supplemental Table 6 – Effect of early therapy on quality of life measures in children with Crohn’s disease**

| **Quality of life measure** | **Early anti-TNF**  **(n = 135; 41%)** | **No early anti-TNF (n = 196; 59%)** | **Total**  **(n = 331)** | **p-value^a^** |
| --- | --- | --- | --- | --- |
| IMPACT-III score at diagnosis, mean (SD) | N = 102/135  61.4 (14.4) | N = 158/196  62.8 (13.1) | N = 260/331  62.3 (13.6) | 0.4 |
| ΔIMPACT-III score by 1 year, mean (SD)^b^ | N = 66/135  +12.7 (12.6)  **p <0.001** | N = 82/196  +10.0 (12.5)  **p <0.001** | N = 148/331  +11.2 (12.5)  **p <0.001** | 0.2 |
| ΔIMPACT-III score by 2 years, mean (SD)^b^ | N = 27/71  +14.3 (12.3)  **p <0.001** | N = 46/121  +12.2 (15.0)  **p <0.001** | N = 72/192  +12.9 (14.1)  **p <0.001** | 0.5 |
| EQ-5D-5L VAS score at diagnosis, mean (SD) | N = 103/135  65.4 (20.5) | N = 152/196  67.0 (19.7) | N = 255/331  66.4 (20.0) | 0.5 |
| ΔEQ-5D-5L VAS score by 1 year, mean (SD) ^b^ | N = 70/135  +17.4 (21.9)  **p <0.001** | N = 91/196  +9.8 (21.5)  **p <0.001** | N = 161/331  +13.1 (22.0)  **p <0.001** | **0.03** |
| ΔEQ-5D-5L VAS score by 2 years, mean (SD) ^b^ | N = 28/71  +13.7 (23.9)  **p = 0.005** | N = 56/121  +12.8 (21.2)  **p <0.001** | N = 84/192  +13.1 (22.0)  **p <0.001** | 0.9 |

^a^ independent two-sample t test; ^b^ paired sample t test.

VAS: visual analogue scale (range 0-100).

**Supplemental Table 7 – Effect of early therapy on treatment targets at two years in children with Crohn’s disease**

| **Outcome** | **Early anti-TNF**  **(n = 71; 37%)** | **No early anti-TNF (n = 121; 63%)** | **Total**  **(n = 192)** | **p-value** |
| --- | --- | --- | --- | --- |
| SSFR, n (%) | 13/66 (20) | 12/114 (11) | 25/180 (14) | 0.09 |
| SSFR*, n (%) | 13/66 (20) | 8/114 (7) | 21/180 (12) | **0.011** |
| SSFMI, n (%) | 39/61 (64) | 33/106 (31) | 72/167 (43) | **<0.001** |
| SSFMI*, n (%) | 36/61 (59) | 19/106 (18) | 55/167 (33) | **<0.001** |
| NCR, n (%) | 39/64 (63) | 63/100 (63) | 102/161 (63) | 0.91 |
| Mucosal healing (as estimated by MINI index), n (%) | 16/28 (57) | 26/48 (54) | 42/76 (55) | 0.80 |

Abbreviations: NCR, normal CRP remission (remission with CRP < 0.5 mg/dl); SSFR(*), sustained steroid-free remission (*without treatment intensification); SSFMI(*), sustained steroid-free mild or inactive disease (*without treatment intensification); MINI-index, mucosal inflammation non-invasive index.

**Supplemental Table 8 – Effect of early therapy on treatment targets at three months in children with Crohn’s disease**

| **Outcome** | **Early anti-TNF**  **(n = 135; 41%)** | **No early anti-TNF (n = 196; 59%)** | **Total**  **(n = 331)** | **p-value** |
| --- | --- | --- | --- | --- |
| Remission, n (%) | 74/134 (55) | 87/182 (48) | 161/316 (57) | 0.19 |
| SFR, n (%) | 70/134 (52) | 80/184 (44) | 150/318 (47) | 0.12 |
| NCR, n (%) | 58/129 (45) | 51/172 (30) | 109/301 (36) | **0.006** |
| NFR, n (%) | 14/80 (18) | 9/124 (7) | 23/204 (11) | **0.02** |
| Small response, n (%) | 104/132 (79) | 117/178 (66) | 221/310 (71) | **0.012** |
| Moderate response, n (%) | 71/132 (54) | 53/178 (30) | 124/310 (40) | **<0.001** |
| FCP response, n (%) | 21/26 (81) | 17/36 (47) | 38/62 (61) | **0.007** |
| Mucosal healing (as estimated by MINI-index) | 23/44 (52) | 16/64 (25) | 39/108 (36) | **0.004** |

Abbreviations: FCP, faecal calprotectin; NCR, normal CRP remission (remission with CRP < 0.5 mg/dl); NFR, normal FCP remission (remission with FCP < 250 mcg/g), SFR, steroid-free remission; MINI-index, mucosal inflammation non-invasive index.

**Supplemental Table 9 – Effect of early therapy on treatment targets at one and two years in children with Crohn’s disease in steroid-free remission or steroid-free mild/inactive disease at 3 months**

| **Outcome** | **Early anti-TNF** | **No early anti-TNF** | **Total** | **p-value** |
| --- | --- | --- | --- | --- |
| SSFR* at 1y for those in SFR at 3 months,  n (%) | 39/66 (59) | 26/75 (35) | 65/141 (46) | **0.004** |
| SSFR* at 2y for those in SFR at 3 months,  n (%) | 13/35 (37) | 8/40 (20) | 21/75 (28) | 0.099 |
| SSFMI* at 1y for those in SFMI at 3 months | 88/105 (84) | 58/127 (46) | 146/232 (63) | **<0.001** |
| SSFMI* at 2y for those in SFMI at 3 months | 36/51 (71) | 19/68 (28) | 55/119 (46) | **<0.001** |

Abbreviations: SFR, steroid-free remission; SSFR*, sustained steroid-free remission without treatment intensification; SFMI; steroid-free mild or inactive disease; SSFMI*, sustained steroid-free mild or inactive disease without treatment intensification.
